# A study to assess the factors associated with severity of road traffic injuries in the emergency medicine department in rural India

**DOI:** 10.1186/1865-1380-8-S1-O1

**Published:** 2015-04-22

**Authors:** Jeedhu Radha

**Affiliations:** 1 Pesimr Hospital, Kuppam, Andhra Pradesh, India

## Objective

To assess the factors associated with severity of road traffic injuries in a rural setting and to compare between the factors and the severity of road traffic injuries.

## Method

Data was collected for patients coming to the emergency department of PESIMSR, Kuppam with the history of road traffic accidents. The study was a descriptive study with prospectve sampling durng the period from October 2012 to April 2014. Data were collected as per the Performa-date and time of accident, date and time of arrival to emergency department, age, sex, education, place of accident, type of vehicle involved, victim involved, usage of seat belts or helmets, type of road, animal barricade, driving licence, alcohol influence and Injury Severity Score.

## Results

During the study period, 750 patients were enrolled. The findings of the study showed that male patients contributed to 83% of the accidents while females were 17% with the mean age between 36 to 38 years for both the sexes. Majority of the patients were below primary school (59.7%). Between the time of accident to the time of arrival to the hospital the mean was 15.52 minutes. With regard to the place of accident, rural area contributed to 85%. The 2-wheeler contributed to 42% among the type of vehicles with rider contributing to 39% under the victim category.

Usage of seat belts or helmets was almost non-existent at 0.4% Most of the roads were single roads (94.4%). The usage of driving license contributed to only 45%. Alcohol consumption in road traffic accidents contributed to 48%. Injury Severity Score showed that 43% of the population in the study was critically ill. The p-value is significant for type of road, area of accident, alcohol influence and usage of seatbelts/helmets.

## Conclusion

Road traffic injury has emerged as a major public health problem which can be definitely prevented and controlled. It places an undue burden on the health care system which is struggling to cope up with prevailing disease burden of communicable diseases and other non-communicable diseases. Efforts need to be made in all areas concerned with road safety, enforcement, education and emergent care.

